# Added-Value Chemicals from Lignin Oxidation

**DOI:** 10.3390/molecules26154602

**Published:** 2021-07-29

**Authors:** Carina A. Esteves Costa, Carlos A. Vega-Aguilar, Alírio E. Rodrigues

**Affiliations:** 1 Laboratory of Separation and Reaction Engineering—Laboratory of Catalysis and Materials (LSRE-LCM), Department of Chemical Engineering, Faculty of Engineering, University of Porto, Rua Dr. Roberto Frias s/n, 4200-465 Porto, Portugal; carina.costa@fe.up.pt (C.A.E.C.); up201700038@edu.fe.up.pt (C.A.V.-A.); 2 Centro de Investigação de Montanha−CIMO, Instituto Politécnico de Bragança, Campus de Santa Apolónia, 5300-253 Bragança, Portugal

**Keywords:** biorefineries, lignocellulosic biomass, lignin, depolymerization, oxidation, phenolic monomers, vanillin and syringaldehyde, dicarboxylic acids

## Abstract

Lignin is the second most abundant component, next to cellulose, in lignocellulosic biomass. Large amounts of this polymer are produced annually in the pulp and paper industries as a coproduct from the cooking process—most of it burned as fuel for energy. Strategies regarding lignin valorization have attracted significant attention over the recent decades due to lignin’s aromatic structure. Oxidative depolymerization allows converting lignin into added-value compounds, as phenolic monomers and/or dicarboxylic acids, which could be an excellent alternative to aromatic petrochemicals. However, the major challenge is to enhance the reactivity and selectivity of the lignin structure towards depolymerization and prevent condensation reactions. This review includes a comprehensive overview of the main contributions of lignin valorization through oxidative depolymerization to produce added-value compounds (vanillin and syringaldehyde) that have been developed over the recent decades in the LSRE group. An evaluation of the valuable products obtained from oxidation in an alkaline medium with oxygen of lignins and liquors from different sources and delignification processes is also provided. A review of C_4_ dicarboxylic acids obtained from lignin oxidation is also included, emphasizing catalytic conversion by O_2_ or H_2_O_2_ oxidation.

## 1. Introduction

Lignocellulosic biomass, including hardwood, softwood, and herbaceous crops, is an abundant renewable resource mainly composed of cellulose, hemicellulose, and lignin [1,2]. The typical lignin content may vary from 18 to 33% in softwoods, 15 to 30% in hardwoods, and 5 to 30% in herbaceous crops [2,3]. However, most biorefineries are currently focused on the valorization of cellulose and hemicellulose, a so-called sugar-based platform. In this context, lignin is usually considered as a low-value residual product and has significant potential as a renewable resource to produce bio-based materials, fuels, and valuable chemicals [4].

In the literature, the main lignin valorization strategies are focused on the depolymerization of lignin into valuable compounds that could be used as platform molecules for industry. The oxidative depolymerization of lignin could be performed through different types of oxidants, and the characteristics of each oxidant determine their activity and selectivity in the oxidation reaction [5]. Therefore, oxidant selection is based on the properties that allow obtaining the maximum yields of the required products. Valuable low molecular weight phenolic compounds, such as vanillin and syringaldehyde, are frequently obtained as the main depolymerization products from lignin oxidation in alkaline medium using oxygen as an oxidant and are the focus of many research works. Vanillin (V, 4-hydroxy-3-methoxybenzaldehyde) is the most commonly produced aroma compound worldwide, with an annual production of 20,000 tons, 15% of which comes from lignin, and only 40 to 50 tons per year are produced from natural vanilla extract [6]. Around 85% of the world supply is produced from petro-based intermediates, especially guaiacol [7]. Vanillin is used as a flavoring and fragrance ingredient in the food or cosmetic industries. It is also an essential intermediate for synthesizing fine chemicals such as pharmaceuticals, e.g., L-DOPA (L-3,4-dihydroxyphenylalanine) [3,8]. Syringaldehyde (S, 4-hydroxy-3,5-dimethoxybenzaldehyde) is a valuable starting material for the chemical and pharmaceutical industries, being a precursor for the synthesis of 3,4,5-trimethoxybenzaldehyde [3,9]. This aromatic aldehyde is also a precursor for the food and cosmetic industry, and it has been synthesized from gallic acid, pyrogallol, and V itself [8,10]. 

In addition to the phenolic product conversion, dicarboxylic acids have also been obtained from the aromatic ring cleavage of lignin or its fragments using more potent oxidants and/or more severe reaction conditions [11]. Dicarboxylic acids such as muconic acid, maleic acid, succinic acid, and malonic acid are valuable platform chemicals and intermediates used in the polymer, pharmaceutical, and food industries [12,13,14]. C_4_ dicarboxylic acids were selected as one of the 12 building blocks for a future bio-based economy, receiving particular attention in recent years [15]. Commercial dicarboxylic acids are all produced from petroleum-based feedstocks or fermentation of edible biomass [12,14]. Usually, severe oxidations have low selectivity, requiring further purification steps. However, their yields show a significant dependence on the type of lignin, extraction techniques, and operating conditions (e.g., pressure, temperature, lignin concentration, and stirring rate). Catalyst presence is also essential to achieve a high yield of a specific acid [12,16,17]. The development of a producing pathway of C_4_ dicarboxylic acids from lignin will arouse interest in chemical industries and biorefineries. 

This work provides a brief overview of lignin oxidation research, emphasizing the work performed by the research group in the Laboratory of Separation and Reaction Engineering (LSRE), University of Porto, Portugal. A strong emphasis is given to lignin oxidation under an alkaline medium with oxygen to produce aromatic compounds, such as aldehydes and acids, comparing different lignin sources, delignification processes, and oxidation technologies. It also includes recent advances on lignin depolymerization towards C_4_ dicarboxylic acids, mainly by catalytic oxidation.

## 2. Lignin: Valorization, Structure, and Classification

Lignin, along with cellulose and hemicellulose, is one of the principal components of the lignocellulosic biomass, accounting for up to 40% of dry biomass weight. A biorefinery concept that integrates processes and technologies for biomass conversion demands an efficient utilization of all three components. Since lignin is the largest non-carbohydrate component in biomass and composed of aromatic compounds, its utilization can significantly enhance the cost competitiveness of the biomass biorefinery. For effective lignin applications, recent biorefinery and lignin valorization developments have tried to fractionate lignin selectively from other components with minimal structural modifications. A new strategy has emerged in the past few years, named the “lignin-first” approach [18,19]. This strategy considers lignin disassembly prior to carbohydrate valorization, through the combination of lignocellulose fractionation with integrated lignin depolymerization [18,19,20,21]. However, a lack of information still exists between the selective and efficient application of this approach in native lignin and the utilization of the obtained compounds in the production of value-added products, thereby influencing the overall economic feasibility of lignocellulosic biorefineries. Consequently, most of the biorefinery schemes are focused on the utilization of easily convertible fractions, while lignin remains relatively under-valorized concerning its potential [22]. 

Nowadays, it has been estimated that 0.5–3.6 billion tons of lignin are produced annually in nature [23]. As the largest chemical process that utilizes plant biomass as raw material, the pulp and paper industry generates 60 million tons of lignin each year [23]. More than 98% of the generated lignin is burned as a source of energy, primarily in the paper and pulp industry, and only 2% of the produced lignin is utilized for commercial macromolecular applications (polyurethane foams, epoxy resins, dispersing or emulsifying agents, and as an additive for concrete and rubber) [24,25]. Apart from its valorization as a polymer or material, lignin can undergo depolymerization reactions towards valuable low molecular weight compounds. However, lignin depolymerization and valorization remain a challenge. 

**Figure 1:**
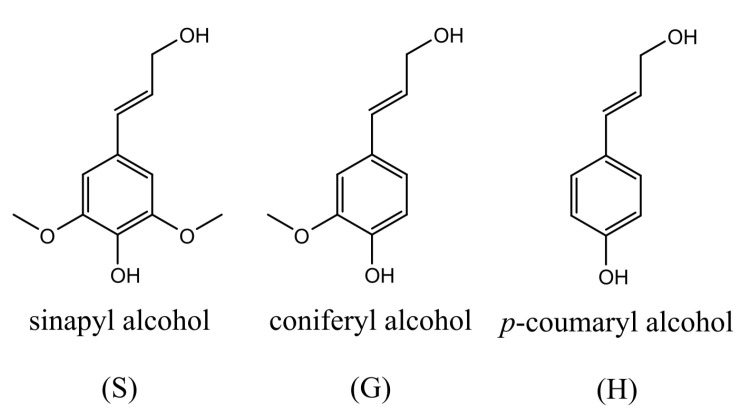
Sinapyl, coniferyl, and p-coumaryl alcohols, precursors of S, G, and H units, respectively.

The lack of established processes that add value to lignin can be attributed mainly to its chemical recalcitrance and complex and heterogeneous composition and structure. Adding to this complexity, the lignin structure is highly dependent on the type of plant and species, the delignification process, and the depolymerization method applied [2,26]. Lignin is a complex three-dimensional amorphous and highly branched aromatic polymer constituted of methoxylated phenylpropane units. Its crucial function in woody biomass is to provide strength, rigidity, and resistance to degradation [2]. There are three primary monomers, syringyl (S), guaiacyl (G), and *p*-hydroxyphenyl (H), derived from the monolignols *p*-coumaryl, coniferyl, and sinapyl alcohols (Figure 1). Depending on the biomass source, lignin varies in the monomer composition. Herbaceous crops contain all three monomers and are relatively rich in H units. Gymnosperm lignins, isolated from softwoods, lack S units, while angiosperm lignins, isolated from hardwoods, are rich in G and S.

**Figure 2:**
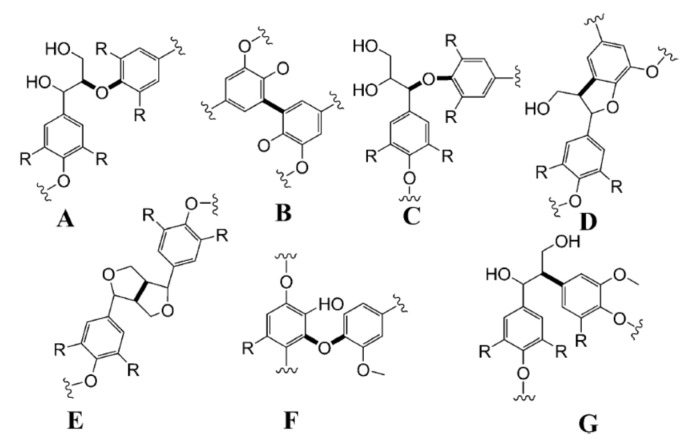
Main structural moieties in lignin structure: (**A**) β-*O*-4, (**B**) 5-5, (**C**) α-*O*-4, (**D**) β-5, (**E**) β-β, (**F**) 4-*O*-5, and (**G**) β-1. (Reprinted from [22] Copyright 2011, with permission from John Wiley and Sons.)

The lignin polymerization process leads to a variety of inter-unit linkages, including aryl ether bonds (β-*O*-4, α-*O*-4, 4-*O*-5) and carbon-carbon linkages (5-5′, β-5, β-1, β–β) [25,27]. The frequency of each type of linkage in lignin’s structure depends on the relative contribution of each monomer to the polymerization process. In native lignin, the most abundant dilignol linkage is the β-*O*-4 type (structure A, Figure 2), accounting for more than 50% of the interunit linkages in lignin structure [8,28]. Therefore, the β-*O*-4 content of isolated lignins strongly depends on the separation method used and the severity of the applied process conditions.

The existence of various interunit linkages, high propensity for the formation of condensed structures when thermochemically processed, poor product selectivity, and ease of use as a solid fuel are the major barriers to the development of lignin-based biorefining technologies [28]. For these reasons, valorization of technical lignins requires detailed insight into the structure and composition impact of the type of plant and species, the delignification process, and the isolation method applied [2].

**Figure 3:**
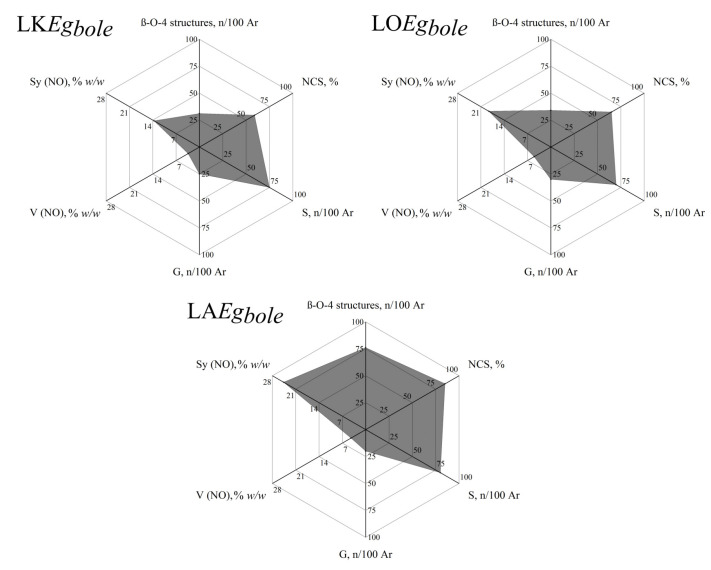
Radar classification for eucalyptus bole lignins produced by different processes (LKEgbole—lignin from industrial Kraft liquor of eucalyptus bole; LOEgbole—lignin produced by ethanol organosolv process of eucalyptus bole; LAEgbole—lignin isolated by mild acidolysis from eucalyptus bole). (Reprinted with permission from [26]. Copyright 2015 American Chemical Society.)

The LSRE lignin research group established a classification tool for lignins based on the major structural characteristics of lignin that allows evaluating lignin relative to its viability as a source of added-value low molecular phenolic compounds, such as vanillin and/or syringaldehyde [26,29,30]. Radar plots represent an effective classification technique for lignins and are a useful approach for assessing their characteristics to maximize lignin valorization [26]. This classification tool combines the assessment of crucial structural characteristics such as H:G:S ratio, condensation, and β-*O*-4 content and allows a qualitative prediction of the yield expected by oxidative depolymerization of different lignins under similar reaction conditions. These characteristics are the descriptors used to build the radar plot for each studied lignin, reducing the unavoidable complexity of lignin structure to its key aspects while maintaining the scientific basis of the data sets with quantitative information [26]. In the works developed in LSRE, the radar plot was built to describe and compare the potential of different lignins to produce vanillin and/or syringaldehyde by oxidative depolymerization with oxygen in an alkaline medium [26,29,30]. The key characteristics selected are the contents in β-*O*-4 structures, non-condensed structures (NCS), S and G units, and the yield of vanillin and syringaldehyde obtained by nitrobenzene oxidation (Figure 3).

The radar classification presented allows the screening of lignins resulting from industrial or preindustrial processes for their potential as a source of valuable phenolic compounds. Considering lignin’s current availability in the side streams of pulp industries and biorefineries, this could be an important approach given lignin exploitation for high added-value applications, improving the economic viability of the plant. 

## 3. Oxidative Depolymerization of Lignin

Considering the vast literature about lignin valorization, the major challenge for converting lignin into value-added compounds is the selective bond cleavage during the depolymerization process. While native lignin is highly reactive towards depolymerization, lignin streams isolated from biorefinery processes such as kraft, sulfite, or organosolv pulping are much more recalcitrant as a consequence of the structural condensation and/or degradation that takes place during the biorefinery process [4,26]. Structural degradation involves cleavage of labile ether and ester linkages (mainly the β-*O*-4 ether bond) and formation of stable carbon-carbon linkages through condensation [25,28,31]. The cleavage of carbon-carbon linkages is the big challenge of lignin depolymerization. This type of linkage is significantly more resistant and most of them remain in lignin’s structure regardless of the depolymerization process, having a strong influence on lignin reactivity. Consequently, products derived from lignin depolymerization strongly depend on the depolymerization method itself but also on the lignin isolation process and the lignin source [2,23,26].

**Figure 4:**
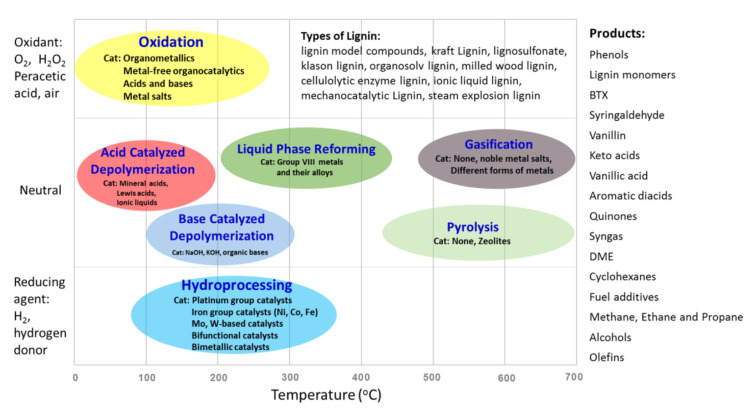
Processes for the conversion of lignin (the abscissa represents the typical temperature range of the lignin conversion processes) (reprinted with permission [36]. Copyright 2015 American Chemical Society).

Various thermal- and chemical-based lignin depolymerization processes have been proposed and applied in the literature [1,22,32,33,34,35,36,37,38,39,40]. Pyrolysis (thermolysis), gasification, hydrogenolysis, hydrolysis under supercritical conditions, and oxidation reactions are the major depolymerization methods studied (Figure 4).

Despite all the depolymerization methods, oxidation aroused great interest in the field of lignin valorization since it represents an effective method for added-value compound production from different sources of lignin [35,40,41,42,43,44,45,46]. Oxidative conversion gives a complex mixture of products highly dependent on the nature of the raw material and the selected reaction conditions. Among them, it is possible to find oligomeric products, phenolic, and non-phenolic compounds. In most studies, the authors focused their attention on producing valuable platform chemicals, including low molecular weight phenolic compounds, with high selectivity and yields. Moreover, the formation of dicarboxylic acids and quinone structures have also been commonly observed as products from the oxidative depolymerization of lignin or its fragments [42]. The activity and selectivity of the oxidative depolymerization of lignin depend on the type and characteristics of the oxidant and the severity of the reaction conditions [5]. Nitrobenzene, some metal oxides (copper (II) oxide), and oxygen (with or without catalyst), all of them in alkaline medium, are mild oxidants that preserve lignin’s aromatic ring and produce mainly aldehydes [22,45]. Nitrobenzene is an effective oxidant that gives the highest product yield. Still, it is an expensive and harmful chemical, and its reduction products are difficult to separate from the reaction medium [47]. However, nitrobenzene is frequently employed for characterization purposes and as a reference in lignin oxidation since it allows one to estimate the maximum conversion of lignin into functionalized phenolics [39,48,49]. In this perspective, some authors have suggested that the yields obtained by NO are about 40–50% of the yield of oxidation with O_2_ in an alkaline medium [39,50]. The use of oxygen in lignin depolymerization is advantageous when economic and environmental questions are considered [48]. This is an inexpensive and green oxidant, which preserves the lignin aromatic rings during the oxidation reaction and presents a high efficiency per weight of oxidant [8,51]. 

**Figure 5:**
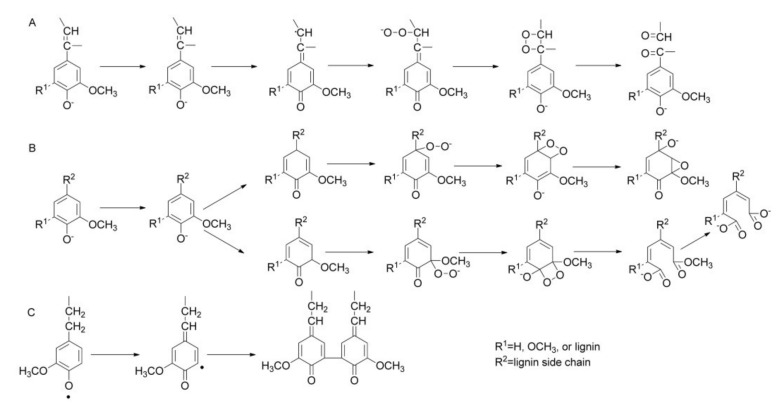
Oxygen oxidation of lignin mechanisms: (**A**) conjugated side-chain oxidation, (**B**) aromatic ring cleavage, and (**C**) oxidative condensation. (Reprinted from [42] Copyright 2015, with permission from John Wiley and Sons.)

Lignin oxidation using oxygen has been extensively studied in the recent decades concerning depolymerization of condensed lignin substrates such as lignosulfonates and kraft lignins. Oxygen delignification proceeds predominantly through a radical chemistry mechanism that plays an essential role in producing functionalized aromatics (Figure 5). Since oxygen is a weak oxidizing agent in its normal state, the reaction requires basic conditions to ionize free phenolic hydroxyl groups in lignin units [1,42]. Consequently, when aromatic products are targeted, the oxidation is mainly performed aerobically in aqueous alkaline (usually NaOH) medium since this enables the selectivity production of phenolic aldehydes such as vanillin and syringaldehyde [40].

### 3.1. Phenolic Compounds from Lignin Oxidation

The history behind utilizing lignin as a source of valuable phenolic compounds can be dated back to the mid-twentieth century as part of the paper industry’s search for new valorization pathways for lignin [52,53]. However, it is well known that the structural transformations and chemical treatments suffered by lignin have a considerable influence on the formation of phenolic compounds since modified lignin has less availability of phenolic precursors in its structure. Alkaline oxidation converts lignin into a complex mixture of products that could be phenolic monomers, dimers, and oligomers. As already stated, the selectivity and efficiency of oxidative depolymerization depend strongly on processing conditions and lignin origin. Alkaline oxidation of softwood lignins produces mainly vanillin and vanillic acid, while syringaldehyde and syringic acid are obtained from hardwood lignins. A literature overview of some representative works about alkaline oxidations of lignin using oxygen shows values in the range of 4–12% *w*/*w* lignin for vanillin and 5–20% *w*/*w* lignin for syringaldehyde, depending on the origin, type, and processing of each lignin [8,22,39,43,44,48,49,54].

**Figure 6:**
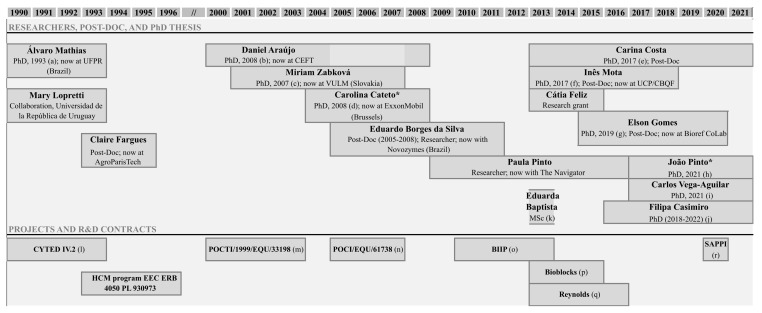
Research in lignin valorization started by Professor Alírio Rodrigues at LSRE (* thesis supervised by Prof. M. Filomena Barreiro (IPB, Portugal)). (**a**) Álvaro Luiz Mathias (UF Paraná, Brazil), “Production of vanillin from kraft lignin: Kinetics and processes”, 1993; (**b**) Daniel Araújo, “Production of vanillin from lignin present in the Kraft black liquor of the pulp and paper industry”, 2008; (**c**) Miriam Zabkova (Slovakia), “Clean technologies for the purification of wastewaters: adsorptive parametric pumping”, 2007; (**d**) Carolina Cateto, “Lignin-based polyurethanes: synthesis, characterization and applications”, 2008; (**e**) Carina Costa, “Vanillin and syringaldehyde from side streams of pulp & paper industries and biorefineries”, 2017; (**f**) Inês Mota, “Fractionation of syringaldehyde and vanillin from oxidation of lignin”, 2017; (**g**) Elson Gomes, “Development of a continuous process for the production of vanillin and syringaldehyde from kraft black liquor”, 2019; (**h**) João Pinto (IPB), “Development of sustainable polymer solutions”, 2019; (**i**) Carlos Alberto Vega-Aguilar (Costa Rica), “Dicarboxylic acids from lignin”, 2021; (**j**) Filipa Casimiro (IPB), “Studies on lignin oxidation and degradation of phenolic products”, 2022; (**k**) Eduarda Baptista, “Ultrafiltração de extrato de casca de Eucalyptus globulus para recuperação de compostos polifenólicos”, 2013; (**l**) CYTED IV.2—“Transformación de lignina en produtos de alto valor agregado”; (**m**) POCTI/1999/EQU/33198—“Development of an integrated process for the production of vanillin from the black liquor of the pulp industry”; (**n**) POCI/EQU/61738/2004—“Purification of vanillin from the lignin oxidation broth”, 2007; (**o**) BIIPP (SI IDT—11551/2010)—“Biorefinaria Integrada na Indústria da Pasta e Papel”; (**p**) BIOBLOCKS—“Concepção de produtos de base biológica como precursores para a bioindústria de síntese química e de biomateriais a partir de fontes lenhocelulósicas”; (**q**) FEUP/RJR/2014/01—Production of Vanillin from tobacco biomass lignin (R. J. Reynolds Tobacco Company, USA); (**r**) Collaboration with SAPPI—“Lignosulphonate’s characterization”, 2020.

Lignin valorization is a solid research field, of more than 30 years, at the Laboratory of Separation and Reaction Engineering (LSRE) (Figure 6). The LSRE group has vast experience in studying alkaline oxidation using oxygen to produce added-value phenolic compounds from lignin, namely vanillin and syringaldehyde. The potential of several lignins and liquors from different sources of biomass and delignification processes was evaluated through batch [26,39,41,43,44,48,55] and/or continuous experiments [56,57,58]. 

**Figure 7:**
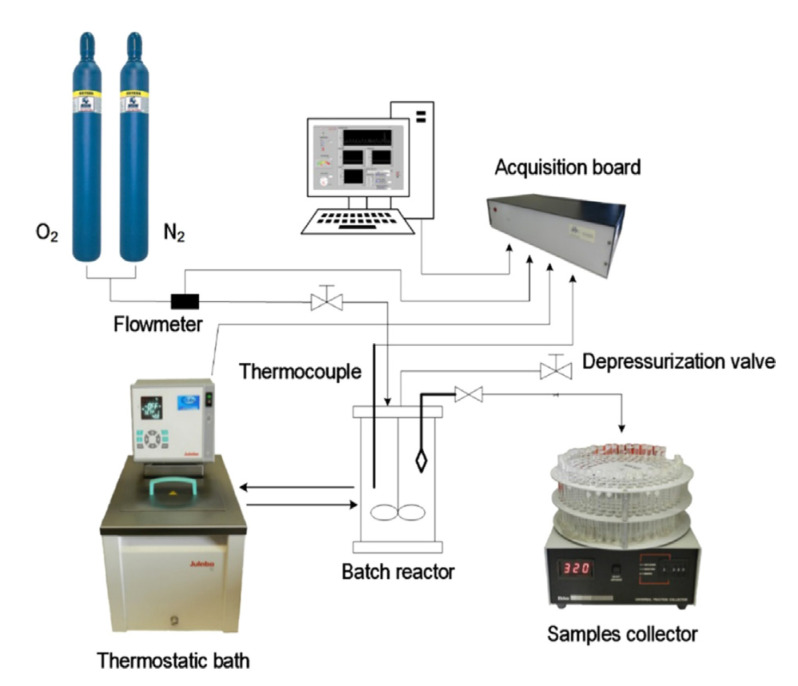
Experimental setup used in batch oxidation experiments at LSRE. (Reprinted with permission from [59]. Copyright 2019 American Chemical Society.)

The batch oxidation experiments were performed in a jacketed reactor with a capacity of 1 L with initial temperature and pressure control at the beginning of the reaction. During the oxidation, the system’s total pressure was kept constant through the continuous feeding of oxygen to the reactor, and the reaction mixture (solution of lignin in NaOH with a selected concentration) was maintained under stirring. The experimental setup used for batch oxidation experiments at LSRE is presented in Figure 7.

The effect of one or several process parameters on lignin oxidative depolymerization performed through batch experiments have been intensively investigated with the primary objective of achieving the ideal conditions for obtaining the maximum conversion of lignin while avoiding the oxidation of the phenolic monomers produced [8,41,43,47,48,54,55,59]. The origin, composition, and processing of lignin (pulping process, isolation method, pretreatment, etc.), the oxygen partial pressure, the initial temperature, and the lignin and sodium hydroxide concentration in the reaction solution were studied, and its effect on the selectivity and efficiency of alkaline oxidative depolymerization process was evaluated [8,48,51,56,59,60]. It was found that a high partial pressure of oxygen reduces reaction time but leads to an increased rate of vanillin oxidation. Increasing the oxygen pressure accelerated both product formation and degradation, and therefore shortened the time needed to reach the maximum product yields [43,61]. In similar works, Schutyser and coworkers found that lignin oxidation under an inert atmosphere produced mainly oligomeric products, while the same reaction under oxygen primarily generated monomeric products [46]. Concerning the effect of pH of the mixture, it was concluded that during the lignin oxidation process, the yield of vanillin decreased when the pH value began to decrease. Moreover, there was a smaller vanillin degradation for strong alkaline conditions that increased significantly when pH was smaller than 11.5 [41,47]. Consequently, high alkali concentrations (pH > 12) are needed to reduce vanillin degradation. For temperature, it was found that an increase in this reaction condition can shorten the reaction time but, on the other hand, results in faster degradation of the aldehydes produced. However, Pacek and coworkers verified that even if usual reaction temperatures were 150–170 °C, the alkaline hydrolysis reaction, caused by the high temperature and strongly alkaline conditions, started at around 120 °C [62]. These authors also argued that hydrolysis started at just above 100 °C, and it produced not only vanillin but also vanillic acid, traces of acetovanillone, and other compounds. Finally, the lignin itself is a variable with a huge influence on the final yields of oxidation products. Considering lignin content in the reaction medium, Fargues et al. found that the vanillin yield only increased for lignin concentration of up to 60 g/L, decreasing for higher values [48]. Moreover, a lignin with a low molecular weight and a less condensed structure tends to give better oxidation results, the presence of residual sugars is highly unfavorable, and the fewer structural transformations or chemical treatments lignin suffers, the better the reactivity toward oxidation and consequently the better the yields of phenolic compounds obtained [39,45,47]. 

**Figure 8:**
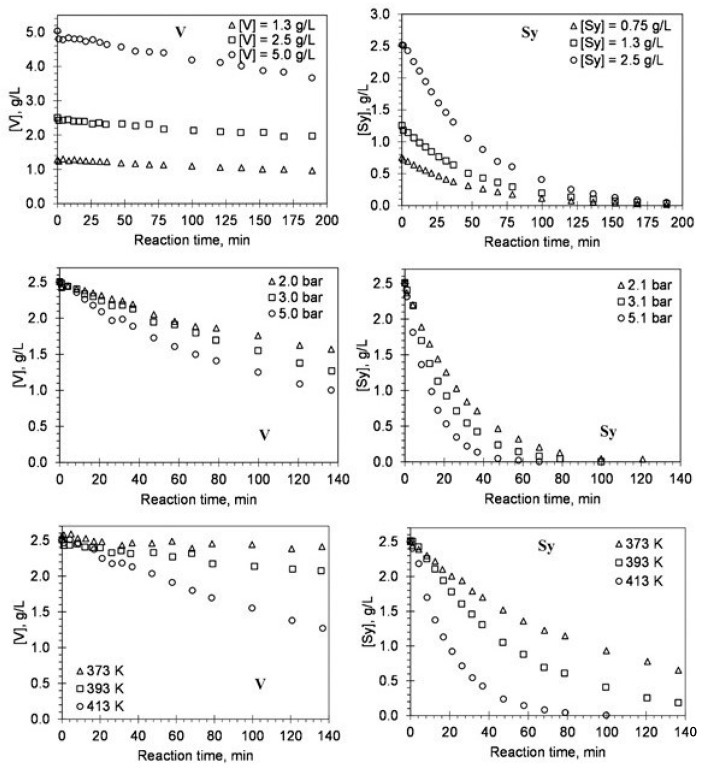
Effect of initial concentration, oxygen partial pressure, and temperature on the degradation as a function of reaction time for V and Sy. The lines represent the fitted kinetic model (experimental conditions: [NaOH] = 80 g/L; p_t_ = 9.8 bar) (reprinted with permission from [59]. Copyright 2019 American Chemical Society).

Using the experimental results from evaluating the main reaction conditions’ effect in the alkaline oxidation with oxygen in a batch reactor, the authors developed a kinetic study of vanillin production [48,56,59,61]. The objective was to measure the reaction orders regarding lignin, oxygen, and vanillin species, as well as the influence of temperature on the kinetic rate constants to discuss the overall process of lignin oxidation [48]. The mathematical model proposed by the authors showed to be able to predict the behavior of vanillin oxidation for the different operating conditions tested. Since the vanillin produced by lignin oxidation is also oxidized and depends on the pH and the temperature of the solution, the influence of these two parameters on the kinetics of vanillin degradation had been studied on vanillin alone. The validation of a kinetic model for vanillin degradation separately confirmed that its degradation can be well predicted in the lignin oxidation experiments [56]. More recently, the kinetic model developed for vanillin degradation was improved by evaluating the degradation of all the main phenolic monomers produced from lignin oxidation: vanillin, vanillic acid, acetovanillone, syringaldehyde, syringic acid, and acetosyringone [59]. The kinetic study considering these individual products is of great interest since; during oxidation, their formation can be simultaneously accompanied by their degradation process that is dependent on the applied oxidation conditions. In Figure 8, the effect of initial concentration, oxygen partial pressure, and temperature on the degradation as a function of reaction time for vanillin (V) and syringaldehyde (Sy) is shown [59].

All the performed studies allowed us to confirm that a trade-off between enhancing the phenolics conversion and minimizing oxidation is achieved for a temperature of around 120 °C, oxygen partial pressure around 3 bar, and lignin concentration of 60 g/L, prepared in a solution of 2 N NaOH [2,8,43,48]. Having these conditions as a starting point for the oxidative depolymerization of lignin, the LSRE team studied the potential of a wide variety of lignins from different origins and delignification processes. The yields of vanillin and syringaldehyde achieved for each lignin by nitrobenzene oxidation and alkaline oxidation with oxygen in the batch reactor are summarized in Table 1. In addition to vanillin and syringaldehyde, other phenolics such as vanillic acid, acetovanillone, syringic acid, and acetosyringone were also identified in minor quantities; their occurrence also has a significant role in the study of reaction efficiency. Moreover, the presented data allow the evaluation of the benefit of lignin source and isolation on product yields.

**Figure 9:**
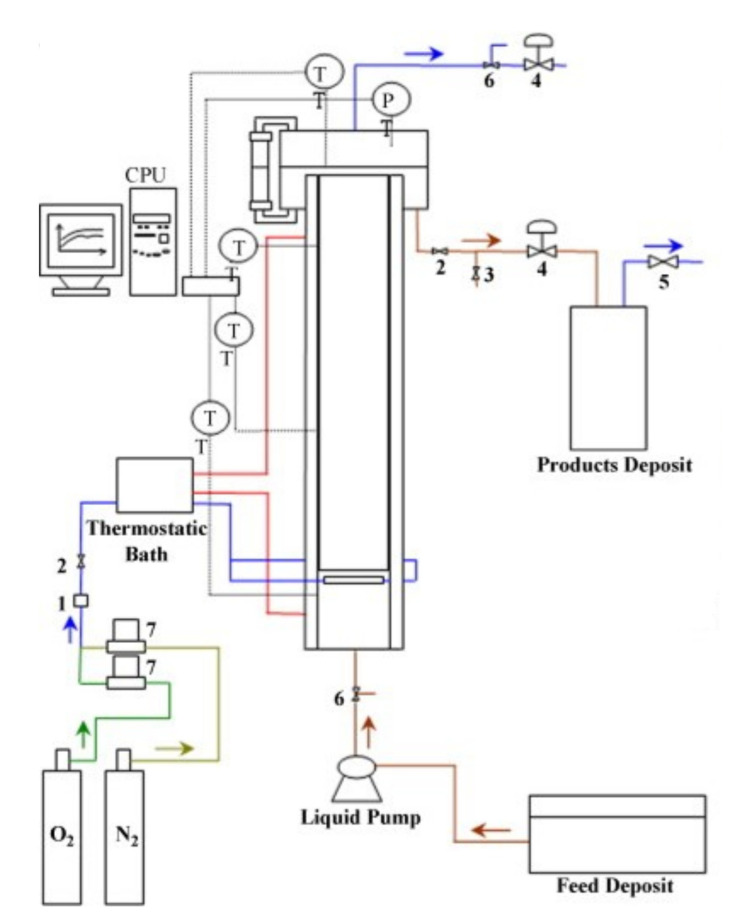
Schematic diagram of the pilot setup for the continuous production of phenolic monomers from lignin: (1) safety valve; (2) on–off valve; (3) electromagnetic valve; (4) needle valve; (5) safety valve; (6) three-way valve; (7) mass flow controller; PT—pressure transducer; TT—thermocouple. (Reprinted from [55] Copyright 2009, with permission from Elsevier.)

Most of the works focused on lignin oxidation have been performed in batch mode. However, from an industrial point of view, the continuous process of lignin oxidation presents more advantages due to the large volumes of liquor generated, the easier control of the process, and the lower overall investments and operating costs [55]. Araújo [56] built an experimental pilot setup to promote lignin oxidation in a continuous operating mode. The schematic diagram of the pilot installation is shown in Figure 9. The bubble column reactor was made in 316L stainless steel with 8 L capacity, and the gas–liquid reaction takes place in the cylindrical body of the reactor. It has a 10 cm internal diameter and 70 cm height and is filled with three modules of Mellapak 750.Y structured packing (Sulzer Chemtech, Switzerland) that enhance the system’s overall mass transfer performance.

However, oxidation in the continuous reactor showed that the lignin conversion was substantially lower than that obtained for the batch process [56,57,58]. To improve the performance of the continuous reactor and reach the production yields obtained in bath mode, some studies focused on the influence of the main reaction were performed [55,56,60]. The results showed that the oxygen mass transfer from the gas phase to the reaction medium of sodium hydroxide and lignin was the limiting step to vanillin formation, and the use of pure oxygen in the gas feed was considered. In this case, the liquid residence time was decreased as the oxygen mass transfer rate increased to avoid excessive vanillin oxidation. A value of vanillin yield, in the exit stream, of approximately 85% of the maximum value obtained in the batch reactor was achieved considering the improvements in the continuous reaction [56,60].

### 3.2. Dicarboxylic Acids from Lignin Oxidation

**Figure 10:**
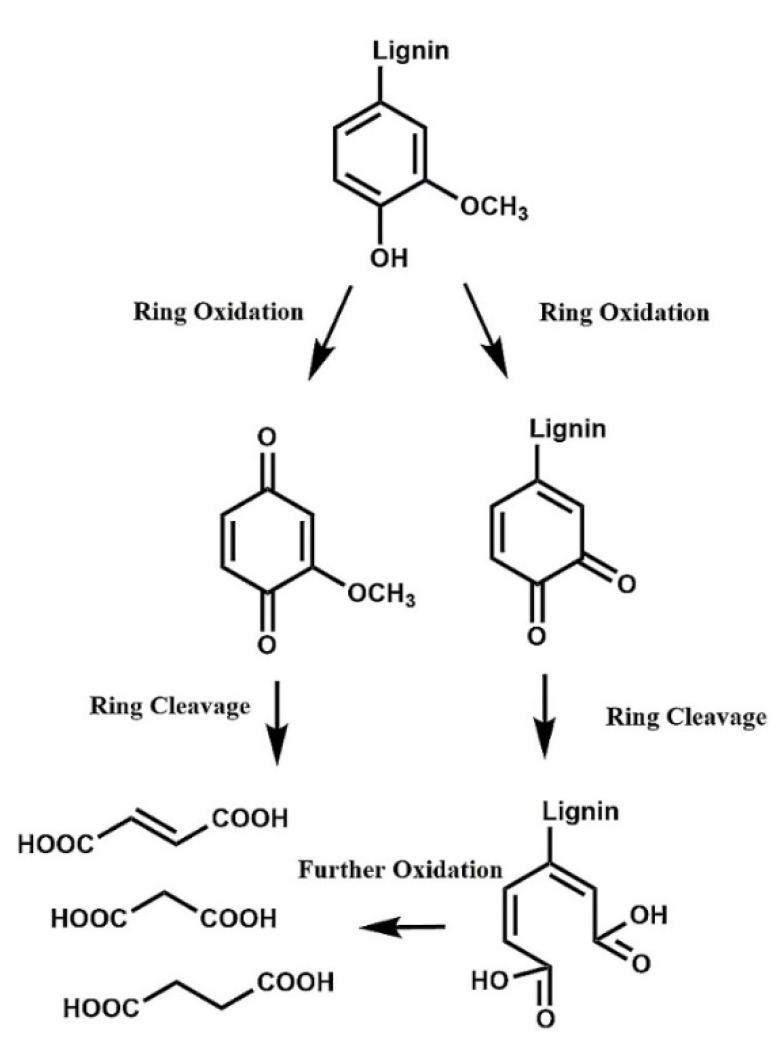
Oxidation of lignin through ring-opening reactions to yield C_4_ dicarboxylic acids. (Reprinted from [64] Copyright 2018, with permission from Elsevier.)

A harsh depolymerization causes cleavage of the remaining bonds that were not broken in mild depolymerization [11]. When the aromatic ring is cleaved (Figure 10), C_6_ acids are obtained (mainly muconic acid), which are quickly degraded to lower carbon-content acids (C_2_-C_4_ acids) [12]. The products can be completely mineralized to CO_2_ and H_2_O under very harsh conditions. Even though C_6_ acids have important industrial uses, they are very unstable and are swiftly converted to C_4_ dicarboxylic acids (C_4_-DCA), which are relatively stable and can be easily separated at the end of the reaction.

The acids with a higher prevalence in lignin oxidation are succinic (SA), malic (MAL), and maleic acids (MA), with small amounts of fumaric (FA) and tartaric (TA) acids. Most of these acids are currently used in food, pharmaceutical, and polymer industries, as well as chemical precursors for 1,4-butanediol, tetrahydrofuran, and γ-butyrolactone [13,14,15,65,66,67,68,69,70]. In the last 15 years, several authors have studied C_4_-DCA from lignin and lignin model compounds under catalytic and non-catalytic conditions, using different strong oxidants, i.e., O_2_, O_3_, H_2_O_2,_ and peracetic acid. Some of these works were focused on lignin depolymerization towards aromatic monomers and reported the C_4_-DCA as a degradation product. Yet, this information is valuable to identify possible research lines and optimal conditions for lignin depolymerization. 

#### 3.2.1. Non-Catalytic Harsh Oxidation

Even though O_2_ is widely used for lignin depolymerization to phenolics, it can cause ring-opening reactions. However, its oxidant power is lower than other oxidants, being less effective towards C_4_-DCA [16,36,71,72]. Demesa et al. [71] performed alkali lignin oxidation using O_2_, achieving up to 3 wt% of succinic acid (SA). Ozone, a stronger oxidant, was used on pyrolytic lignin, obtaining a small amount of SA (2.0 wt%) and maleic acid (MA) (2.3 wt%) [73]. Comparatively, previous works from other research groups using O_3_ on technical lignins showed low yields of C_4_-DCA [74,75].

Hydrogen peroxide has received a strong focus when ring-opening reactions are the objective because it is more reactive than O_2_, with the benefit of being environmentally benign, allowing milder conditions, and avoiding mass transfer barriers that appear between liquid and gas phases [22,76,77,78]. However, given that H_2_O_2_ is a weak acid, its reactivity is strongly associated with the pH, being stable at acidic conditions but decomposing in alkaline conditions [79,80]. Lignin model compounds (guaiacol, syringol, and phenol) were oxidized using H_2_O_2_ at 300 °C and short times, obtaining different C_1_-C_6_ dicarboxylic acids [81]. Catechol oxidation reached very high yields of TA, FA, and MAL, with up to 41% C_4_-DCA [79]. Both studies concluded that the oxidation of the phenolic model compounds goes through *o*-benzoquinones and *p*-benzoquinones, yielding muconic and 2,5-dioxo-3-hexenoic acids, which are highly unstable and are degraded to C_4_-DCA. Some C_4_-DCA were identified in hardwood kraft lignin oxidation using peracetic acid [82]. Flow reactor oxidation of alkali lignin using H_2_O_2_ showed up to 13 wt% SA [83] when using very high temperatures and short times. SA was formed above 150 °C, confirming that harsh conditions are required to achieve valuable yields, at least in a non-catalyzed reaction.

Following the interest in the C_4_-DCA obtained from lignin, the LSRE group analyzed the peroxide oxidation of lignin and lignin model compounds [84], studying the effect of the methoxy substituents in the lignin aromatic ring on the production of C_4_-DCA. It was found that methoxy substituents increased reactivity toward peroxide oxidation, causing lignin model compounds with more methoxy substituents (syringic acid) to be degraded easily, while *p*-hydroxybenzoic acid (without methoxy substituents) was more resistant to oxidation. Compounds with lower methoxy substituents (*p*-hydroxybenzoic and vanillic acid) showed higher overall C_4_-DCA yield and higher succinic acid (SA) yield than syringic acid. Interestingly, when two lignins with different S:G ratios were compared, the hardwood lignin (higher S:G ratio) not only showed a better lignin conversion but also achieved a higher SA yield (3.2 wt%). The softwood lignin achieved a lower SA yield (2.5 wt%) but a higher MAL yield in the first minutes of the reaction. This study demonstrated that even though the methoxy substituent can reduce C_4_-DCA production from model compounds, they boost lignin reactivity towards peroxide oxidation, making it easier to depolymerize lignin into smaller fragments that will be converted to C_4_-DCA, increasing their final yield.

#### 3.2.2. Catalytic Lignin Harsh Oxidation

Peroxide oxidation of lignin towards C_4_-DCA can be performed using different types of catalyst, which can vary from expensive noble metals, cheaper zeolites, or even homogeneous metal ions, such as Fenton’s reagent [71]. More than 65% of the latest publications on lignin conversion to C_4_-DCA use catalytic conversion, and those works were focused on two oxidants: O_2_ and H_2_O_2_, with the latter having a higher amount of research. 

Homogeneous catalysts are mainly transition metal ions with at least two oxidation states, e.g., Cu^+/2+^ and Fe^2+/3+^. Oxygen oxidation with these catalysts produced very low C_4_-DCA yields [85,86]. With H_2_O_2_ in Fenton’s conditions, phenol oxidation yielded 8% of MA [87], and other model compounds produced small amounts of MA and FA (<2%) [88]. However, no C_4_-DCA was obtained when lignin was oxidized [89], concluding that Fenton’s catalyst approach is not efficient for depolymerization towards C_4_-DCA.

Different heterogeneous catalysts have been used with distinct outcomes. Perovskite-type oxides (such as chalcopyrite) have in their structures transition metals with at least two different oxidation states, which catalyzes H_2_O_2_ oxidation [42,90]. Chalcopyrite (CuFeS_2_) presented promising results in model compounds [12] and biorefinery lignins (diluted-acid corn stover lignin: 7% SA, 1% MAL), but with low SA yields for bagasse lignin [91]. Chalcopyrite nanoparticles used on lignin at acidic pH produced high yields of SA (12%) with low yields of FA and MA (1%, each) [92]. Other catalysts, such as sodium percarbonate in alkaline conditions, yielded ~1% SA and MA/FA traces for bagasse oxidation [91]. Gas-phase O_2_ oxidation using aluminium-vanadium-molybdenum oxide and vanadium pyrophosphate in a fluidized bed only produced small amounts of MA (1.5 wt%) [93], while eight supported metal catalysts (involving V, Mo, Mg, and W) produced a small amount of C_4_-DCA (SA and/or MA/FA) [94]. It was V-W/HZSM-5 that produced the highest yields (nearly 2% SA and 12% MA/FA), confirming that V^5+^ activates the aromatic rings in monomeric units.

**Figure 11:**
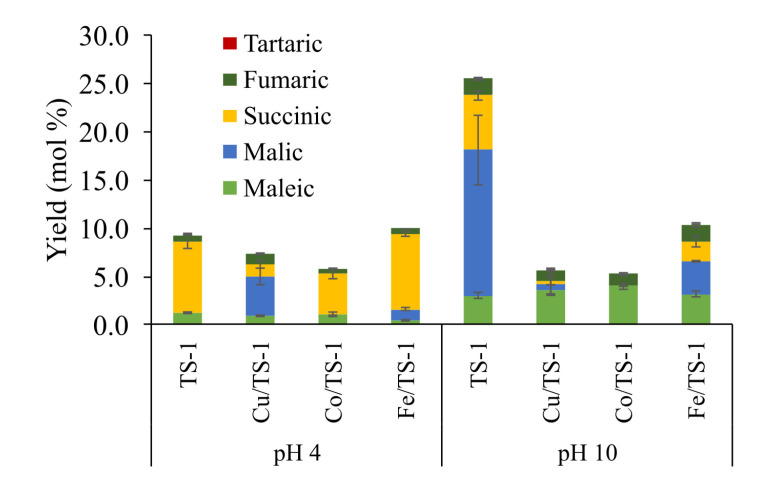
Effect of TS-1 and modified TS-1 catalysts in the C_4_-DCA yields for the wet peroxide oxidation of vanillic acid. (Modified from [101]. Copyright 2020, with permission from Elsevier.)

Titanium silicalite 1 (TS-1), a zeolite with an MFI structure and no more than 3% of TiO_2_ in its structure, has hydrophobic properties that permit peroxide oxidation of non-polar compounds in an aqueous medium. The H_2_O_2_ is adsorbed in the Ti tetrahedral sites to form Ti-OOH groups that act as the active species, increasing the overall reactivity. Currently, it is used for cyclohexanone ammoximation to caprolactam and the production of propylene oxide [95,96,97]. Guaiacol peroxide oxidation using TS-1 in mild alkaline conditions achieved high percentages of MA and oxalic acid, with small percentages of FA and MAL [98]. Other works oxidizing furfural reported good yields on MA [99,100]. Following these promising results, the LSRE research group selected this catalyst to evaluate the conversion of lignin and lignin model compounds into C_4_-DCA, with particular attention on SA yield, due to its high value to polymer production and chemical precursor [13,65]. An initial approach by Vega-Aguilar et al. [101] used vanillic acid as a lignin model compound to evaluate different operating conditions under H_2_O_2_ oxidation. Specific C_4_-DCA type and yield were affected by pH, achieving more hydroxylated acids in alkaline pH, while SA was the primary acid in acidic pH (Figure 11). After the optimum reaction time, the acid yields were slowly degraded to low molecular weight compounds, e.g., formic, acetic, and oxalic acids. Additional modifications of TS-1 catalyst were made with Fe, Cu, and Co oxides by wet impregnation due to the observed catalytic effects of these transition metal oxides. Only the Fe/TS-1 catalyst increased SA yield slightly in acidic pH. Oxygen oxidation of vanillic acid was also tested using TS-1 in a Büchi reactor, but no promising results were obtained.

The TS-1 catalyst was used for oxidation of different industrial lignins (Indulin AT (*IAT*), Alkaline lignin (*ALK*), Lignol (*EOL*) lignin, and *E. globulus* kraft lignin (*EKL*)). In this work, Vega-Aguilar et al. [102] evaluated different operating conditions and the catalyst reusability in several cycles. The C_4_-DCA showed a strong dependence on pH, temperature, reaction time, catalyst load, and H_2_O_2_ load. The main C_4_-DCA were MAL and SA, and there was an increase in the SA yield between the non-catalyzed and the catalyzed reactions, up to four times for indulin AT and alkaline lignins (Table 2). High temperatures were needed to achieve a good lignin conversion and C_4_-DCA yields, but acid degradation also happened at these temperatures. Acidic and neutral pHs showed the best yields, with the neutral pH being the best operating condition since lignin shows a poor solubility at acidic pH. A 10 wt% H_2_O_2_ loading was the optimum amount; a higher amount over-oxidized the products, while a lower one avoided complete conversion. Interestingly, the best reaction parameters were similar to vanillic acid oxidation. The catalyst showed stability and a slight C_4_-DCA yield decrease after five cycles, due to catalyst loss during the centrifugation steps, associated with the small particle size, and not contamination or catalyst degradation. This study showed that TS-1 can be a valuable catalyst to improve SA as the main C_4_-DCA after lignin oxidation, and further studies can be conducted to enhance its use on larger scales. 

As mentioned before, this research line has not only recently been followed in the LSRE, but also worldwide. Therefore, as results are relatively new and the process has not the maturity of the lignin oxidation process for aromatic monomers, this topic has not been included yet in the integrated process developed by Prof. Rodrigues’s research group. 

## 4. Integrated Process

In addition to all the work developed regarding lignin oxidation, the LSRE team has been working on a general concept of an integrated process that combines reaction engineering and efficient separation processes for converting lignin from pulping spent liquors into value-added aldehydes, such as vanillin and syringaldehyde. 

The integrated concept proposed starts with the oxidation of a portion of the by-product streams generated in biorefineries. The pulping liquors or isolated lignins, obtained by acidification/precipitation or ultrafiltration, will be depolymerized through alkaline oxidation with oxygen [41,43,44,48,57]. Then, the oxidized stream continues to an ultrafiltration process, leading to the separation of high molecular weight fraction of degraded lignin from the lower molecular weight species [58,103,104]. Vanillin and syringaldehyde go preferentially to the permeate stream due to their low molecular weights, while the oxidized high molecular weight fraction of lignin remains in the retentate. The fraction retained by the membrane during the ultrafiltration process, the retentate, can be considered as a raw material for lignin-based polyurethanes. The production of polymers from lignin is an attractive approach since it can take advantage of its functional groups and macromolecular proprieties. This application has been the topic of intense research in the Polytechnic Institute of Bragança (IPB), and materials with quite promising properties were already obtained [105,106,107]. 

After a membrane separation step, the permeate, containing the low molecular weight phenolates and excess NaOH, flows through a packed bed with a polymeric resin [58,108]. The separation of the different species will be achieved by adsorption, which can fractionate the permeate solution in families of chemicals, namely phenolic acids, aldehydes, and ketones [109,110]. In the end, the phenolic compounds of interest, aldehydes in this case, that are present in the enriched desorbed fraction will be recovered by crystallization [60].

**Figure 12:**
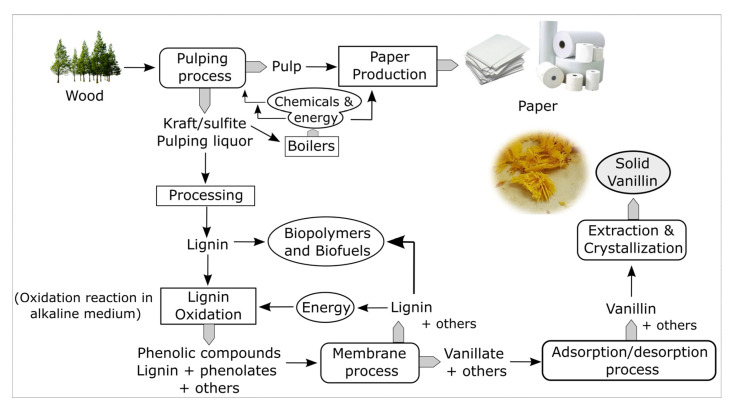
Integrated process to produce value-added phenolics and polymers from lignin in a biorefinery concept. (Modified from [55] Copyright 2009, with permission from Elsevier.)

This complete process (reaction and separation steps), represented in Figure 12, could be integrated into a pulp and paper industrial plant, considering the possibility of part of the lignin from side streams (spent liquor) to be deviated to produce high added-value chemicals instead of only being burned to generate energy. Moreover, this process perfectly fits into the scope of new emerging lignocellulosic-based biorefineries concerning lignin valorization.

## Figures and Tables

**Table 1:** Yields of vanillin and syringaldehyde obtained by nitrobenzene oxidation (NO) and alkaline oxidation using O_2_ of lignins studied at LSRE (experimental conditions of alkaline oxidation using O_2_ and performed in reactor batch: T_i_ = 120 °C; [lignin] = 60 g/L; [NaOH] = 80 g/L; pO_2_ = 3 bar; p_t_ = 9.8 bar).

	Vanillin, % *w*/*w* _lignin_ *		Syringaldehyde, % *w*/*w* _lignin_ *	
	NO **	Alkaline Oxid.	NO **	Alkaline Oxid.
LInAT ^1,2^	9.3	3.4	-	-
LWest ^1^	12.1	4.4	-	-
LBoostS ^1^	11.1	3.1	-	-
LOrgs ^1^	4.8	1.2	13.2	2.5
KL ^3^	2.9	0.73	12.5	1.9
KLlig ^3^	3.4	1.2	13.6	2.8
EKL ^3^	2.2	0.71	9.2	1.4
EKLlig ^3^	2.5	0.82	9.5	2.0
HTEKL ^3^	3.4	0.54	9.8	1.5
HTEKLlig ^3^	2.6	0.94	9.8	2.0
SL ^3^	2.5	1.5	11.3	3.3
LTobO_but_^4^	2.8	0.74	2.5	0.34
LTobO_ethan_^4^	7.2	1.2	4.8	0.94
LCelbi	1.7	0.81	9.5	2.1

* Values reported on dry weight and corrected to non-volatile solid weight after deducting ashes and carbohydrates; ** NO conditions detailed in the literature [63]. ^1^ [39]; ^2^ [56]; ^3^ [44]; ^4^ [26]. (**LInAT**—lignin Indulin AT, industrial pine kraft lignin from Westvăjco; **LWest**—kraft lignin from southern pine (*Pinus* spp.), supplied by Westvăjco; **LBoostS**—kraft lignin from softwood (mainly spruce) isolated by LignoBoost process; **LOrgs**—lignin extracted from beech wood by organosolv process using aqueous ethanol, supplied by Fraunhofer (Germany); **KL**, **EKL**, and **HTEKL**—*Eucalyptus globulus* kraft liquor collected at different stages of a Portuguese bleached kraft pulp plant: at the outlet of kraft digester (KL), after the evaporation stage (EKL), and after heat treatment just before the recovery furnace (HTEKL); **KLlig**, **EKLlig**, and **HTEKLlig**—lignins isolated from kraft liquors KL, EKL, and HTEKL, respectively; **SL**—industrial spent liquor from magnesium-based acidic sulfite pulping of *E. globulus* collected after the evaporation step in a Portuguese sulfite pulp mill; **LTobO_but_** and **LTobO_ethan_**—lignin from tobacco stalks produced by organosolv process using butanol and ethanol, respectively; **LCelbi**—kraft lignin from hardwood (eucalyptus), supplied by Celbi.).

**Table 2:** Best C_4_-DCA yields for four industrial lignin oxidations for catalyzed and non-catalyzed wet peroxide oxidations using TS-1 catalyst [102].

Lignin	Catalyst	Acid Yields (wt%)				
		Succinic	Malic	Maleic	Fumaric	Tartaric
ALK	No	1.6	1.0	n.d.	n.d.	n.d.
	TS-1	5.8	6.6	0.1	0.04	0.9
IAT	No	2.4	4.8	n.d.	n.d.	n.d.
	TS-1	11.3	10.1	0.1	0.06	n.d.
EOL	No	6.9	22.6	n.d.	n.d.	n.d.
	TS-1	9.7	19.5	0.1	0.04	n.d.
EKL	No	0.6	3.6	n.d.	n.d.	n.d.
	TS-1	7.6	5.5	n.d.	n.d.	n.d.

n.d.: not detected.

## References

[1] Gillet S., Aguedo M., Petitjean L., Morais A.R.C., da Costa Lopes A.M., Łukasik R.M., Anastas P.T. (2017). Lignin transformations for high value applications: Towards targeted modifications using green chemistry. Green Chem..

[2] Rodrigues A.E., Pinto P.C.d.R., Barreiro M.F., da Costa C.A.E., da Mota M.I.F., Fernandes I. (2018). An Integrated Approach for Added-Value Products from Lignocellulosic Biorefineries.

[3] Strassberger Z., Tanase S., Rothenberg G. (2014). The pros and cons of lignin valorisation in an integrated biorefinery. RSC Adv..

[4] Vishtal A.G., Kraslawski A. (2011). Challenges in industrial applications of technical lignins. BioResources.

[5] Lin S., Dence C., Lin S.Y., Dence C.W. (1992). Methods in Lignin Chemistry.

[6] Bomgardner M.M. (2014). Following many routes to naturally derived vanillin. Chem. Eng. News.

[7] Huang W.-B., Du C.-Y., Jiang J.-A., Ji Y.-F. (2013). Concurrent synthesis of vanillin and isovanillin. Res. Chem. Intermed..

[8] Pinto P.C.R., Borges da Silva E.A., Rodrigues A.E., Baskar C., Baskar S., Dhillon R.S. (2012). Lignin as source of fine chemicals: Vanillin and syringaldehyde. Biomass Conversion: The Interface of Biotechnology, Chemistry and Materials Science.

[9] Erofeev Y.V., Afanas’eva V.L., Glushkov R.G. (2004). Synthetic routes to 3,4,5-trimethoxybenzaldehyde (review). Pharm. Chem. J..

[10] Ibrahim M.N.M., Sriprasanthi R.B., Shamsudeen S., Adam F., Bhawani S.A. (2012). A Concise Review of the Natural Existance, Synthesis, Properties, and Applications of Syringaldehyde. BioResources.

[11] Rinaldi R., Jastrzebski R., Clough M.T., Ralph J., Kennema M., Bruijnincx P.C.A., Weckhuysen B.M. (2016). Paving the Way for Lignin Valorisation: Recent Advances in Bioengineering, Biorefining and Catalysis. Angew. Chemie-Int. Ed..

[12] Ma R., Guo M., Zhang X. (2014). Selective Conversion of Biorefinery Lignin into Dicarboxylic Acids. ChemSusChem.

[13] Gérardy R., Debecker D.P., Estager J., Luis P., Monbaliu J.-C.M. (2020). Continuous Flow Upgrading of Selected C 2 –C 6 Platform Chemicals Derived from Biomass. Chem. Rev..

[14] Kamm B., Gruber P.R., Kamm M. (2008). Biorefineries-Industrial Processes and Products: Status Quo and Future Directions. CHEMISTRY Int..

[15] Werpy T., Petersen G. (2004). Top Value Added Chemicals from Biomass: Volume I--Results of Screening for Potential Candidates from Sugars and Synthesis Gas..

[16] Kang S., Li X., Fan J., Chang J. (2013). Hydrothermal conversion of lignin: A review. Renew. Sustain. Energy Rev..

[17] Asgari F., Argyropoulos D.S. (1998). Fundamentals of oxygen delignification. Part II. Functional group formation/elimination in residual kraft lignin. Can. J. Chem..

[18] Korányi T.I., Fridrich B., Pineda A., Barta K. (2020). Development of ‘Lignin-First’ Approaches for the Valorization of Lignocellulosic Biomass. Molecules.

[19] Sun Z., Cheng J., Wang D., Yuan T.-Q., Song G., Barta K. (2020). Downstream Processing Strategies for Lignin-First Biorefinery. ChemSusChem.

[20] Chen L., van Muyden A.P., Cui X., Fei Z., Yan N., Laurenczy G., Dyson P.J. (2021). Lignin First: Confirming the Role of the Metal Catalyst in Reductive Fractionation. JACS Au.

[21] Renders T., Van Den Bosch S., Koelewijn S.F., Schutyser W., Sels B.F. (2017). Lignin-first biomass fractionation: The advent of active stabilisation strategies. Energy Environ. Sci..

[22] Pandey M.P., Kim C.S. (2011). Lignin Depolymerization and Conversion: A Review of Thermochemical Methods. Chem. Eng. Technol..

[23] Gellerstedt G., Henriksson G., Belgacem M.N., Gandini A. (2008). Chapter 9-Lignins: Major sources, structure and properties. Monomers, Polymers and Composites from Renewable Resources.

[24] Chan J.C., Paice M., Zhang X. (2020). Enzymatic oxidation of lignin: Challenges and barriers toward practical applications. ChemCatChem.

[25] Constant S., Wienk H.L.J.J., Frissen A.E., de Peinder P., Boelens R., van Es D.S., Grisel R.J.H.H., Weckhuysen B.M., Huijgen W.J.J.J., Gosselink R.J.A.A. (2016). New insights into the structure and composition of technical lignins: A comparative characterisation study. Green Chem..

[26] Costa C.A.E.E., Pinto P.C.R., Rodrigues A.E. (2015). Radar Tool for Lignin Classification on the Perspective of Its Valorization. Ind. Eng. Chem. Res..

[27] Calvo-Flores F.G., Dobado J.A. (2010). Lignin as renewable raw material. ChemSusChem.

[28] Berlin A., Balakshin M., Gupta V.K., Tuohy M.G., Kubicek C.P., Saddler J., Xu F. (2014). Industrial Lignins. Bioenergy Research: Advances and Applications.

[29] Costa C.A.E., Coleman W., Dube M., Rodrigues A.E., Pinto P.C.R. (2016). Assessment of key features of lignin from lignocellulosic crops: Stalks and roots of corn, cotton, sugarcane, and tobacco. Ind. Crops Prod..

[30] Costa C.A.E.E., Pinto P.C.R.R., Rodrigues A.E. (2018). Lignin fractionation from E. Globulus kraft liquor by ultrafiltration in a three stage membrane sequence. Sep. Purif. Technol..

[31] Costa C.A.E.E., Pinto P.C.R., Rodrigues A.E. (2014). Evaluation of chemical processing impact on E. globulus wood lignin and comparison with bark lignin. Ind. Crops Prod..

[32] Schutyser W., Renders T., Van den Bosch S., Koelewijn S.F., Beckham G.T., Sels B.F. (2018). Chemicals from lignin: An interplay of lignocellulose fractionation, depolymerisation, and upgrading. Chem. Soc. Rev..

[33] Azadi P., Inderwildi O.R., Farnood R., King D.A. (2013). Liquid fuels, hydrogen and chemicals from lignin: A critical review. Renew. Sustain. Energy Rev..

[34] Evstigneyev E.I. (2018). Selective depolymerization of lignin: Assessment of yields of monomeric products. J. Wood Chem. Technol..

[35] Lange H., Decina S., Crestini C. (2013). Oxidative upgrade of lignin-Recent routes reviewed. Eur. Polym. J..

[36] Li C., Zhao X., Wang A., Huber G.W., Zhang T. (2015). Catalytic Transformation of Lignin for the Production of Chemicals and Fuels. Chem. Rev..

[37] Liu X., Bouxin F.P., Fan J., Budarin V.L., Hu C., Clark J.H. (2020). Recent advances in the catalytic depolymerization of lignin towards phenolic chemicals: A review. ChemSusChem.

[38] Patil V., Adhikari S., Cross P., Jahromi H. (2020). Progress in the solvent depolymerization of lignin. Renew. Sustain. Energy Rev..

[39] Rodrigues Pinto P.C., Borges da Silva E.A., Rodrigues A.E. (2011). Insights into Oxidative Conversion of Lignin to High-Added-Value Phenolic Aldehydes. Ind. Eng. Chem. Res..

[40] Vangeel T., Schutyser W., Renders T., Sels B.F. (2018). Perspective on Lignin Oxidation: Advances, Challenges, and Future Directions. Top. Curr. Chem..

[41] Araújo J.D.P., Grande C.A., Rodrigues A.E. (2010). Vanillin production from lignin oxidation in a batch reactor. Chem. Eng. Res. Des..

[42] Ma R., Xu Y., Zhang X. (2015). Catalytic Oxidation of Biorefinery Lignin to Value-added Chemicals to Support Sustainable Biofuel Production. ChemSusChem.

[43] Mathias A.L., Rodrigues A.E. (1995). Production of vanillin by oxidation of pine kraft lignins with oxygen. Holzforschung.

[44] Pinto P.C.R., Costa C.E., Rodrigues A.E. (2013). Oxidation of Lignin from Eucalyptus globulus Pulping Liquors to Produce Syringaldehyde and Vanillin. Ind. Eng. Chem. Res..

[45] Villar J.C.C., Caperos A., García-Ochoa F. (2001). Oxidation of hardwood kraft-lignin to phenolic derivatives with oxygen as oxidant. Wood Sci. Technol..

[46] Schutyser W., Kruger J.S., Robinson A.M., Katahira R., Brandner D.G., Cleveland N.S., Mittal A., Peterson D.J., Meilan R., Román-Leshkov Y. (2018). Revisiting alkaline aerobic lignin oxidation. Green Chem..

[47] Fache M., Boutevin B., Caillol S. (2016). Vanillin production from lignin and its use as a renewable chemical. ACS Sustain. Chem. Eng..

[48] Fargues C., Mathias Á., Rodrigues A. (1996). Kinetics of Vanillin Production from Kraft Lignin Oxidation. Ind. Eng. Chem. Res..

[49] Tarabanko V., Petukhov D. (2003). Study of mechanism and improvement of the process of oxidative cleavage of ligins into the aromatic aldehydes. Chem. Sustain. Dev..

[50] Tarabanko V.E., Fomova N.A., Kuznetsov B.N., Ivanchenko N.M., Kudryashev A. (1995). V On the mechanism of vanillin formation in the catalytic oxidation of lignin with oxygen. React. Kinet. Catal. Lett..

[51] Costa C.A.E. (2017). Vanillin and Syringaldehyde from Side Streams of Pulp and Paper Industries and Biorefineries. Ph.D. Thesis.

[52] Ragauskas A.J., Beckham G.T., Biddy M.J., Chandra R., Chen F., Davis M.F., Davison B.H., Dixon R.A., Gilna P., Keller M. (2014). Lignin valorization: Improving lignin processing in the biorefinery. Science.

[53] Zhang X., Tu M., Paice M.G. (2011). Routes to potential bioproducts from lignocellulosic biomass lignin and hemicelluloses. BioEnergy Res..

[54] Santos S.G., Marques A.P., Lima D.L.D., Evtuguin D.V., Esteves V.I. (2011). Kinetics of Eucalypt lignosulfonate oxidation to aromatic aldehydes by oxygen in alkaline medium. Ind. Eng. Chem. Res..

[55] da Silva E.A.B., Zabkova M., Araújo J.D., Cateto C.A., Barreiro M.F., Belgacem M.N., Rodrigues A.E. (2009). An integrated process to produce vanillin and lignin-based polyurethanes from Kraft lignin. Chem. Eng. Res. Des..

[56] Araújo J.D.P. (2008). Production of Vanillin from Lignin Present in the Kraft Black Liquor of the Pulp and Paper Industry. Ph.D. Thesis.

[57] Araújo J.D.P., Grande C.A., Rodrigues A.E. (2009). Structured packed bubble column reactor for continuous production of vanillin from Kraft lignin oxidation. Catal. Today.

[58] Gomes E.D., Rodrigues A.E. (2019). Lignin biorefinery: Separation of vanillin, vanillic acid and acetovanillone by adsorption. Sep. Purif. Technol..

[59] Casimiro F.M., Costa C.A.E.E., Botelho C.M., Barreiro M.F., Rodrigues A.E. (2019). Kinetics of oxidative degradation of lignin-based phenolic compounds in batch reactor. Ind. Eng. Chem. Res..

[60] Gomes E.D. (2019). Development of a Continuous Process for the Production of Vanillin and Syringaldehyde from Kraft Black Liquor. Ph.D. Thesis.

[61] Fargues C., Mathias Á., Silva J., Rodrigues A. (1996). Kinetics of vanillin oxidation. Chem. Eng. Technol..

[62] Pacek A.W., Ding P., Garrett M., Sheldrake G., Nienow A.W. (2013). Catalytic conversion of sodium lignosulfonate to vanillin: Engineering aspects. Part 1. Effects of processing conditions on vanillin yield and selectivity. Ind. Eng. Chem. Res..

[63] Pinto P.C.R.R., da Silva E.A.B.B., Rodrigues A.E. (2010). Comparative Study of Solid-Phase Extraction and Liquid−Liquid Extraction for the Reliable Quantification of High Value Added Compounds from Oxidation Processes of Wood-Derived Lignin. Ind. Eng. Chem. Res..

[64] Ma R., Guo M., Zhang X. (2018). Recent advances in oxidative valorization of lignin. Catal. Today.

[65] Litsanov B., Brocker M., Oldiges M., Bott M., Bisaria V.S., Kondo A. (2014). Succinic Acid. Bioprocessing of Renewable Resources to Commodity Bioproducts.

[66] de Jong E., Jungmeier G., Elsevier B.V. (2015). Biorefinery Concepts in Comparison to Petrochemical Refineries. Industrial Biorefineries and White Biotechnology.

[67] Cok B., Tsiropoulos I., Roes A.L., Patel M.K. (2014). Succinic acid production derived from carbohydrates: An energy and greenhouse gas assessment of a platform chemical toward a bio-based economy. Biofuels Bioprod. Biorefining.

[68] West T.P. (2017). Microbial Production of Malic Acid from Biofuel-Related Coproducts and Biomass. Fermentation.

[69] Martin-Dominguez V., Estevez J., Ojembarrena F.D.B., Santos V.E., Ladero M. (2018). Fumaric Acid Production: A Biorefinery Perspective. Fermentation.

[70] Goldberg I., Rokem J.S. (2014). Fumaric Acid Biosynthesis and Accumulation. Bioprocess. Renew. Resour. Commod. Bioprod..

[71] Demesa A.G., Laari A., Turunen I., Sillanpää M. (2015). Alkaline Partial Wet Oxidation of Lignin for the Production of Carboxylic Acids. Chem. Eng. Technol..

[72] Cabral Almada C., Kazachenko A., Fongarland P., Da Silva Perez D., Kuznetsov B.N., Djakovitch L. (2020). Oxidative depolymerization of lignins for producing aromatics: Variation of botanical origin and extraction methods. Biomass Convers. Biorefinery.

[73] Figueirêdo M.B., Deuss P.J., Venderbosch R.H., Heeres H.J. (2019). Valorization of Pyrolysis Liquids: Ozonation of the Pyrolytic Lignin Fraction and Model Components. ACS Sustain. Chem. Eng..

[74] Evtuguin D., Robert D. (1997). The detection of muconic acid type structures in oxidized lignins by13C NMR spectroscopy. Wood Sci. Technol..

[75] Quesada J., Rubio M., Gómez D. (1999). Ozonation of Lignin Rich Solid Fractions from Corn Stalks. J. Wood Chem. Technol..

[76] Guélou E., Barrault J., Fournier J., Tatibouët J.M. (2003). Active iron species in the catalytic wet peroxide oxidation of phenol over pillared clays containing iron. Appl. Catal. B Environ..

[77] Cheng C., Wang J., Shen D., Xue J., Guan S., Gu S., Luo K.H. (2017). Catalytic oxidation of lignin in solvent systems for production of renewable chemicals: A review. Polymers.

[78] Bhargava S.K., Tardio J., Prasad J., Föger K., Akolekar D.B., Grocott S.C. (2006). Wet oxidation and catalytic wet oxidation. Ind. Eng. Chem. Res..

[79] Yin G., Jin F., Yao G., Jing Z. (2015). Hydrothermal Conversion of Catechol into Four-Carbon Dicarboxylic Acids. Ind. Eng. Chem. Res..

[80] Xiang Q., Lee Y.Y. (2000). Oxidative cracking of precipitated hardwood lignin by hydrogen peroxide. Appl. Biochem. Biotechnol..

[81] Suzuki H., Cao J., Jin F., Kishita A., Enomoto H., Moriya T. (2006). Wet oxidation of lignin model compounds and acetic acid production. J. Mater. Sci..

[82] Ma R., Guo M., Lin K.T., Hebert V.R., Zhang J., Wolcott M.P., Quintero M., Ramasamy K.K., Chen X., Zhang X. (2016). Peracetic Acid Depolymerization of Biorefinery Lignin for Production of Selective Monomeric Phenolic Compounds. Chem.-A Eur. J..

[83] Hasegawa I., Inoue Y., Muranaka Y., Yasukawa T., Mae K. (2011). Selective Production of Organic Acids and Depolymerization of Lignin by Hydrothermal Oxidation with Diluted Hydrogen Peroxide. Energy Fuels.

[84] Vega-Aguilar C.A., Barreiro M.F., Rodrigues A.E. (2021). Effect of Methoxy Substituents on Wet Peroxide Oxidation of Lignin and Lignin Model Compounds: Understanding the Pathway to C 4 Dicarboxylic Acids. Ind. Eng. Chem. Res..

[85] Rovio S., Kallioinen A., Tamminen T., Hakola M., Leskelä M., Siika-ahoa M. (2012). Catalysed alkaline oxidation as a wood fractionation technique. BioResources.

[86] Wu G., Heitz M. (1995). Catalytic mechanism of Cu2^+^ and Fe3^+^ in alkaline O_2_ oxidation of lignin. J. Wood Chem. Technol..

[87] Faisal I. (2009). Oxidation of Phenolic Wastewater by Fenton’s Reagent. Iraqi J. Chem. Pet. Eng..

[88] Kang J., Irmak S., Wilkins M. (2019). Conversion of lignin into renewable carboxylic acid compounds by advanced oxidation processes. Renew. Energy.

[89] Zeng J., Yoo C.G., Wang F., Pan X., Vermerris W., Tong Z. (2015). Biomimetic fenton-catalyzed lignin depolymerization to high-value aromatics and dicarboxylic acids. ChemSusChem.

[90] Ansaloni S., Russo N., Pirone R. (2018). Wet Air Oxidation of Industrial Lignin Case Study: Influence of the Dissolution Pretreatment and Perovskite-type Oxides. Waste Biomass Valorization.

[91] Cronin D.J., Zhang X., Bartley J., Doherty W.O.S.S. (2017). Lignin Depolymerization to Dicarboxylic Acids with Sodium Percarbonate. ACS Sustain. Chem. Eng..

[92] Bi Z., Li Z., Yan L. (2018). Catalytic oxidation of lignin to dicarboxylic acid over the CuFeS2 nanoparticle catalyst. Green Process. Synth..

[93] Lotfi S., Boffito D.C., Patience G.S. (2015). Gas-Phase Partial Oxidation of Lignin to Carboxylic Acids over Vanadium Pyrophosphate and Aluminum-Vanadium-Molybdenum. ChemSusChem.

[94] Lotfi S., Boffito D.C., Patience G.S. (2016). Gas–solid conversion of lignin to carboxylic acids. React. Chem. Eng..

[95] Clerici M.G. (2015). The Activity of Titanium Silicalite-1 (TS-1): Some Considerations on Its Origin. Kinet. Catal..

[96] Gamba A., Tabacchi G., Fois E. (2009). TS-1 from First Principles. J. Phys. Chem. A.

[97] Xia C., Peng X., Zhang Y., Wang B., Lin M., Zhu B., Luo Y., Shu X., Karamé I. (2017). Environmental-Friendly Catalytic Oxidation Processes Based on Hierarchical Titanium Silicate Zeolites at SINOPEC. Green Chemical Processing and Synthesis.

[98] Su J., Yang L., Liu R.N., Lin H. (2014). Low-temperature oxidation of guaiacol to maleic acid over TS-1 catalyst in alkaline aqueous H2O2 solutions. Chinese J. Catal..

[99] Rodenas Y., Mariscal R., Fierro J.L.G.G., Martín Alonso D., Dumesic J.A., López Granados M. (2018). Improving the production of maleic acid from biomass: TS-1 catalysed aqueous phase oxidation of furfural in the presence of γ-valerolactone. Green Chem..

[100] Alba-Rubio A.C., Fierro J.L.G., León-Reina L., Mariscal R., Dumesic J.A., López Granados M. (2017). Oxidation of furfural in aqueous H2O2 catalysed by titanium silicalite: Deactivation processes and role of extraframework Ti oxides. Appl. Catal. B Environ..

[101] Vega-Aguilar C.A., Barreiro M.F., Rodrigues A.E. (2020). Catalytic wet peroxide oxidation of vanillic acid as a lignin model compound towards the renewable production of dicarboxylic acids. Chem. Eng. Res. Des..

[102] Vega-Aguilar C.A., Barreiro M.F., Rodrigues A.E. (2021). Valorisation of lignin into C4 dicarboxylic acids by catalytic wet peroxide oxidation using TS-1 catalyst. Ind. Crops Prod..

[103] Mota I.F., Pinto P.R., Ribeiro A.M., Loureiro J.M., Rodrigues A.E. (2018). Downstream processing of an oxidized industrial kraft liquor by membrane fractionation for vanillin and syringaldehyde recovery. Sep. Purif. Technol..

[104] Zabkova M., da Silva E.A.B., Rodrigues A.E. (2007). Recovery of vanillin from lignin/vanillin mixture by using tubular ceramic ultrafiltration membranes. J. Memb. Sci..

[105] Cateto C.A., Barreiro M.F., Rodrigues A.E., Brochier-Salon M.C., Thielemans W., Belgacem M.N. (2008). Lignins as macromonomers for polyurethane synthesis: A comparative study on hydroxyl group determination. J. Appl. Polym. Sci..

[106] Cateto C.A., Barreiro M.F., Rodrigues A.E. (2008). Monitoring of lignin-based polyurethane synthesis by FTIR-ATR. Ind. Crops Prod..

[107] Cateto C.A., Barreiro M.F., Rodrigues A.E., Belgacem M.N. (2009). Optimization Study of Lignin Oxypropylation in View of the Preparation of Polyurethane Rigid Foams. Ind. Eng. Chem. Res..

[108] Zabkova M., Otero M., Minceva M., Zabka M., Rodrigues A.E. (2006). Separation of synthetic vanillin at different pH onto polymeric adsorbent Sephabeads SP206. Chem. Eng. Process. Process. Intensif..

[109] Gomes E.D.D., Mota M.I.I., Rodrigues A.E.E. (2018). Fractionation of acids, ketones and aldehydes from alkaline lignin oxidation solution with SP700 resin. Sep. Purif. Technol..

[110] Mota M.I.F., Pinto P.C.R., Loureiro J.M., Rodrigues A.E. (2016). Adsorption of vanillin and syringaldehyde onto a macroporous polymeric resin. Chem. Eng. J..

